# Synthesis and Characterization of Multifunctional Chitosan–Silver Nanoparticles: An In-Vitro Approach for Biomedical Applications

**DOI:** 10.3390/ph17091229

**Published:** 2024-09-18

**Authors:** Gulamnabi Vanti, Naresh Poondla, Prasath Manogaran, Nagappa Teradal, Veeresh S, Ram Kaulgud, Mahantesh Kurjogi

**Affiliations:** 1 Multidisciplinary Research Unit, Karnataka Medical College and Research Institute, Hubli 580021, India; nabisam1@gmail.com (G.V.); vshugar@gmail.com (V.S.); mrukimshubli@gmail.com (R.K.); 2 Department of Neurology, Icahn School of Medicine at Mount Sinai, New York, NY 10029, USA; naresh.poondla@mssm.edu; 3 Center for Global Health Research, Saveetha Medical College& Hospital, Chennai 602105, India; 4 Department of Applied Chemistry, Saveetha School of Engineering, Saveetha Institute of Medical and Technical Science (SIMATS), Chennai 602105, India; 5 Department of Clinical and Translational Sciences, Marshall University, Huntington, WV 25755, USA; prasathbiomed@gmail.com; 6 Department of Chemistry, J. S. S. Arts, Science and Commerce College, Gokak 591307, India; nlteradal@gmail.com

**Keywords:** silver nanoparticles, chitosan, antibacterial activity, antioxidant activity, wound healing activity

## Abstract

Antibiotics are successful in promoting health quality by preventing various infectious diseases and minimizing mortality and morbidity all over the world. However, the indiscriminate use of antibiotics has led to the emergence of multi-drug-resistant bacteria, which pose a serious threat to health care sector. Therefore, it is necessary to develop novel antimicrobial agents with versatile characteristics, such as antibacterial activity, low toxicity, wound healing potency, and antioxidant property. In this context, silver chitosan nanoparticles were synthesized in the present study, and their physical characterization revealed that the size of synthesized chitosan–silver nanoparticles was 14–25 nm, with positive surface charge. The functional groups and crystalline nature of the nanoparticles were confirmed by FT-IR and XRD analysis. Further, the silver chitosan nanoparticles showed antibacterial activity against two important clinical pathogens, *S. aureus* and *E. coli*. The MTT assay carried out in the present study showed that the synthesized nanoparticles are non-toxic to host cells. A scratch assay on fibroblast cells (L292) demonstrated that the silver chitosan nanoparticles showed promising wound healing activity. A fluorescent DCFH-DA staining assay revealed anantioxidant property of the synthesized nanoparticles. Overall, the study emphasizes the versatile nature of synthesized chitosan–silver nanoparticles, suggesting their great compatibility for biomedical applications.

## 1. Introduction

Antibiotics play a crucial role in enhancing health by preventing numerous infectious diseases and reducing mortality and morbidity worldwide. However, their overuse has led to the rise of multi-drug-resistant bacteria, presenting a significant challenge to the health care sector [1,2]. The continued rise in antibiotic-resistant bacteria may be driven by the high frequency of spontaneous mutations and the exchange of genetic material between microbial strains [3]. On the other hand, an inadequate blood circulation and increased inflammation in the wound area complicate the treatment process, especially in surface wound infections. In addition, wound recovery and the wound–healing process may also lead to increased health care expenses. *Staphylococcus aureus* is one of the most common ESKAPE pathogens (*Enterococcus faecium*, *S. aureus*, *Klebsiella pneumoniae*, *Acinetobacter baumannii*, *Pseudomonas aeruginosa*, and *Enterobacter* spp.) that interrupt the wound healing process by biofilm formation [4]. Wound healing is a complex process involving the interaction between immune cells and the extracellular matrix to halt bleeding and prevent infection. Additionally, wounds increase the levels of reactive oxygen species, which further activate transcriptional signals and trigger caspase-3-dependent apoptosis, leading to cell death [5]. However, systemic antibiotics can barely penetrate into wound biofilms and topically applied antibiotics can easily lead to sensitization. Therefore, it is essential to explore novel antimicrobial agents with multifunctional properties, including antimicrobial activity, wound healing capabilities, low toxicity, and excellent compatibility. In this regard, nanotechnology has gathered worldwide interest due to the versatile characteristics of nanoparticles. Additionally, chitosan, a natural biopolymer, has demonstrated significant potential in controlled drug delivery and wound healing. Its biocompatibility and non-toxic nature further enhance its appeal for biomedical applications. In recent years, chitosan-based nanomaterials have been extensively studied for wound healing applications due to their antibacterial properties and biocompatibility. Additionally, chitosan is recognized for its ability to enhance the proliferative phase of wound healing and reduce inflammation by stimulating inflammatory cells, macrophages, and fibroblasts in the wound area [6]. Several researchers have adopted a combination of chitosan and metal nanoparticles in the synthesis of chitosan-based nanoparticles in order to enhance antimicrobial activity and biological safety for clinical application. Numerous studies have explored various strategies for preparing these chitosan-based metal nanoparticles, including hydrogels, fibers, sponges, films, membranes, and scaffolds, all of which are utilized in wound healing assays [7,8,9,10,11,12,13]. Despite numerous reports on various chitosan-based formulations for wound healing assays, several major challenges persist. These include issues with controlled drug delivery, the sterilization of chitosan-based formulations, and the scalability of manufacturing processes for commercial production [14]. Moreover, there is a lack of information regarding the powdered form of chitosan-based silver nanoparticles and their application in wound treatment [15]. Silver, on the other hand, is one of the most extensively studied metals, historically known for its broad-spectrum antimicrobial activity. Silver nanoparticles are able to penetrate bacterial cell walls, disrupt cell membrane structure, and overall improve the healing process, particularly in topical wound infections.

Chitosan and silver each address infection and wound healing through distinct mechanisms. Therefore, it is crucial to develop an effective, biocompatible therapeutic that combines these two agents to leverage their synergistic effects on microbes and the wound healing process. In this study, a novel, cost-effective, and sustainable method was utilized to fabricate silver on chitosan, aiming to exhibit antibacterial properties, enhance wound healing, and scavenge reactive oxygen species.

## 2. Results

### 2.1. Nanoparticles Characterization

**Figure 1:**
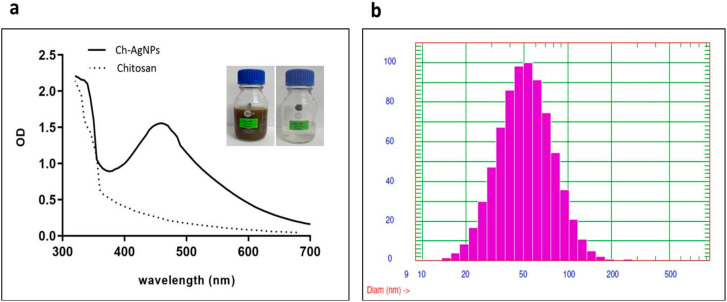
(**a**) The color change and the UV-Vis spectra of synthesized Ch-AgNPs. (**b**) Gaussian distribution analysis of the synthesized Ch-AgNPs.

The reaction conditions and reducing agents used in the preparation of nanoparticles play a crucial role in the synthesis of nanoparticles. In the current study, chitosan and silver nitrate were efficiently reduced to nano size using hydrazine as a reducing agent. A change in color was observed visually and confirmed by an absorption peak at ~455 nm (Figure 1a), suggesting the transition of Ag^0^ from AgNO_3_ substrate. Dynamic Light Scattering (DLS) results revealed that the synthesized NPs were in the range of 16–190 nm, with an average diameter of 55 ± 19.5 nm, by Gaussian distribution (Figure 1b).

**Figure 2:**
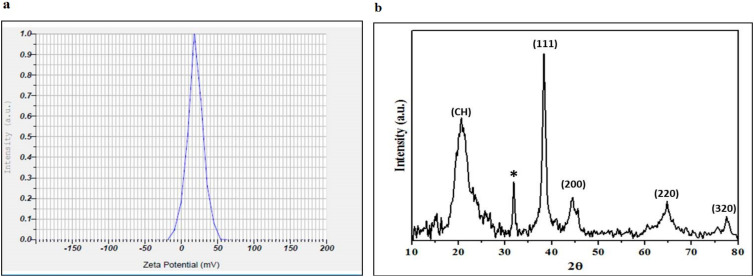
(**a**) The zeta potential of the synthesized nanoparticles (**b**) XRD characterization of synthesized nanoparticles, * Unassigned peak.

Particle size distribution analysis in aqueous solution showed a 0.64 reading, suggesting that the nanoparticles were highly dispersive inaqueous medium. The surface charge of the NPs was confirmed by the zeta potential, which showed a positive (+19.2 mV) surface charge (Figure 2a). Further, the crystal structure was observed by diffraction peaks in XRD at 38.008°, 44.211°, 64.012°, and 78.54°, which correspond to the planes of 111, 200, 220, and 320, respectively, suggesting the simple cubic phase of silver nanoparticles with crystalline nature (JCPDS file no. 89-3722). The broadening of Bragg’s peaks presents the successful formation of Ch-AgNPs. The average crystalline size of silver was calculated using the Scherrer formula, as below:

D = 0.94 λ/βCosϴ

Whereby D is the average crystallite domain size perpendicular to the reflecting planes, λ is the X-ray wavelength, β is the full width at half maximum (FWHM), and ϴ is the diffraction angle.

The average crystalline size was calculated to be 23.82 nm, and unassigned peaks (32.004°) were also observed (marked with asterisks), suggesting that the crystallization of the bio-organic phase occurs on the Ag nanoparticles (Figure 2b).

**Figure 3:**
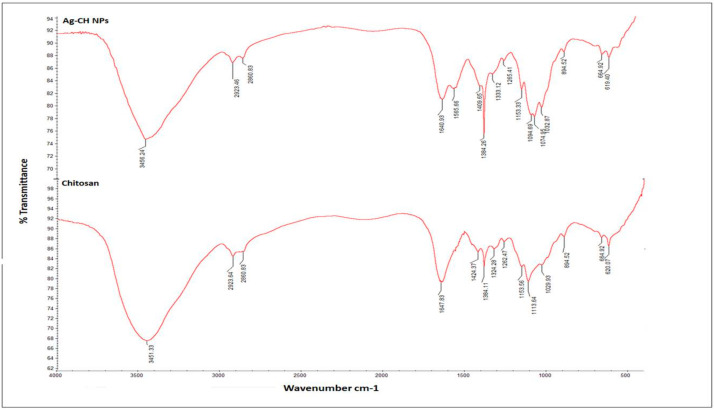
FT-IR spectra of synthesized Ch-AgNPs.

The FT-IR spectra of bulk chitosan alone indicated a band at 3451.33 cm^−1^, which was basically due to the stretching of hydroxyl and amino groups. However, in the same position, more stretching was observed in the NPs with a band length of 3456.24 cm^−1^, which was slightly higher compared to bulk chitosan. Further, NPs witnessed a sharp peak at 1384.26 cm^−1^ and 1640.93 cm^−1^, whereas these peaks were found at 1384.11 cm^−1^ and 1647.83 cm^−1^ in bulk chitosan, suggesting a stretching of the functional groups -CONH_2_ (Figure 3). Other peak variations such as 1500–1000 cm^−1^ in the NPs observed in the present study might be due to –OH, C-O, and C-H bending, which help to stabilize and redistribute the vibrational frequencies in NPs.

### 2.2. Electron Microscopy and EDS Analysis of NPs

**Figure 4:**
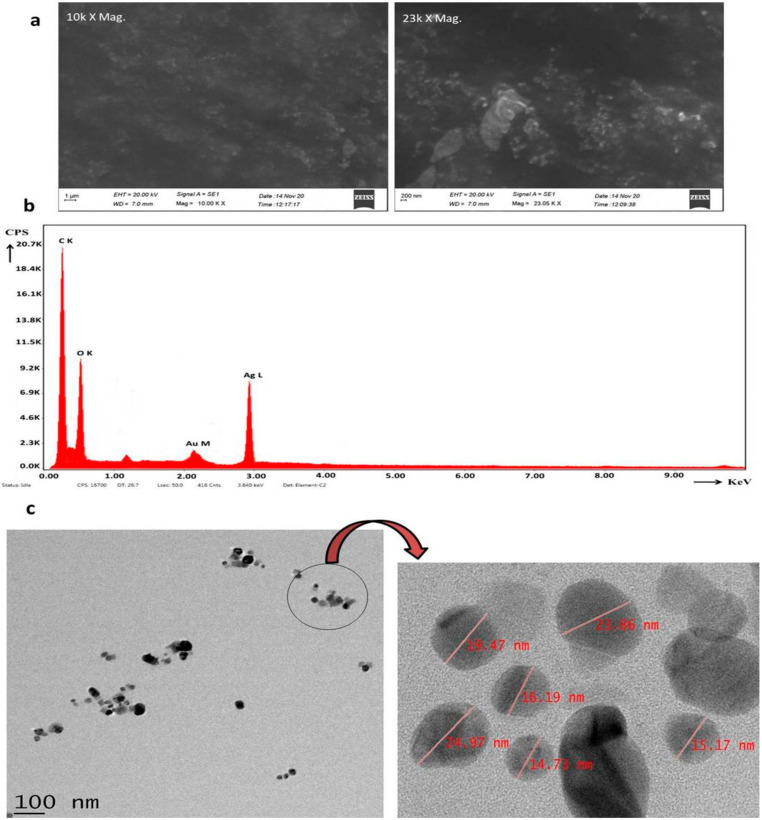
(**a**) Shape and size of synthesized NPs as depicted by SEM. (**b**) Elemental analysis of nano Ag by EDS. (**c**) Morphology confirmation of synthesized nanoparticles by TEM.

Observation under SEM revealed the morphology of the NPs, which were spherical in shape and ranged from 20 to 50 nm in size (Figure 4a). Additionally, X-ray studies showed the presence of the silver element at its 2.98 KeV (Lα) energy level by EDS analysis (Figure 4b). Other observed peaks in the spectra might have been due to deposition during sample preparation. In support of SEM, the morphology of NPs was further validated with TEM, and the analysis confirmed the spherical shape of the NPs; the observed NPs were in the range of 14 to 25 nm in diameter (Figure 4c).

### 2.3. Efficacy of NPs on Clinical Pathogens

**Figure 5:**
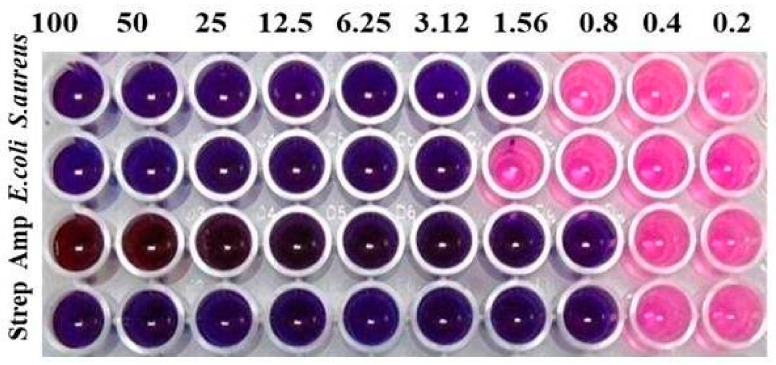
MIC of Ch-AgNPs against *S. aureus* and *E. coli*.

In the present study, the synthesized Ch-AgNPs showed antibacterial activity, with MIC values of 0.8 µg/mL and 1.6 µg/mL against Gram-positive *S. aureus* and Gram-negative *E. coli*, respectively, when compared with that of standard Streptomycin and Ampicillin antibiotics, respectively, with MIC 0.8 µg/mL (Figure 5).

### 2.4. Toxicity and WoundHealing Assay

**Figure 6:**
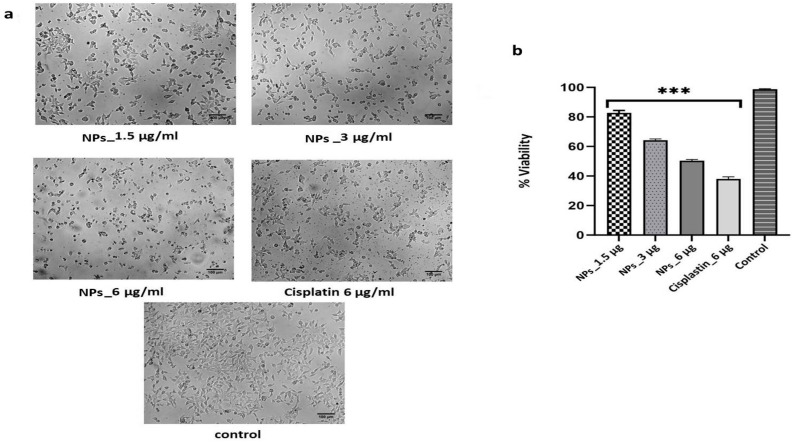
(**a**) Toxicity assay of Ch-AgNPs. (**b**) IC_50_ value of Ch-AgNPs, as determined by the MTT assay *** *p* < 0.01.

The MTT assay showed that HEK-293 cells (NCL Pune, India) were viable up to 82% at 1.5 µg/mL NP concentrations; however, as the concentration of the NPs increased, the HEK-293 cells viability decreased, and at 6 µg/mL of NPs concentration, the HEK-293 cell viability was 51.2 ± 0.9%. On the other hand, the standard drug cisplatin at 6 µg/mL showed 38.1 ± 0.5% HEK-293 cell viability (Figure 6a,b). Furthermore, the 50% inhibitory concentration (IC_50_) of synthesized NP was calculated and estimated to be 5.53 µg/mL.

**Figure 7:**
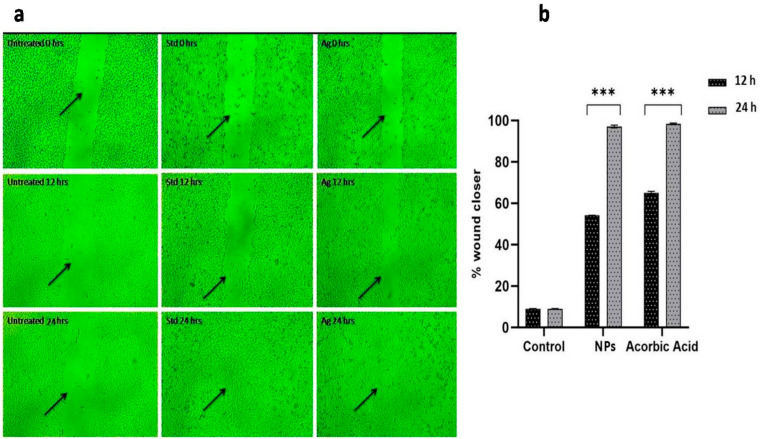
(**a**) Scratch assay depicting wound healing activity of synthesized nanoparticles. (**b**) Graph illustrating 97% wound closure when treated with Ch-AgNPs at 24 h, *** *p* < 0.001.

Further, the wound healing activity of NPs assayed by the artificial scratch assay method on normal fibroblast cells (L292 cells) showed 97% wound closure with 18.9 µM cell migrations at 0.55 µg/mL of NPs. Meanwhile, buffer-incubated cells showed 14% wound closure at 24 h of incubation. However, standard ascorbic acid used in this study showed 98.8% wound healing activity when tested on fibroblast cells (Figure 7a,b).

### 2.5. Antioxidant Activity of NPs

**Figure 8:**
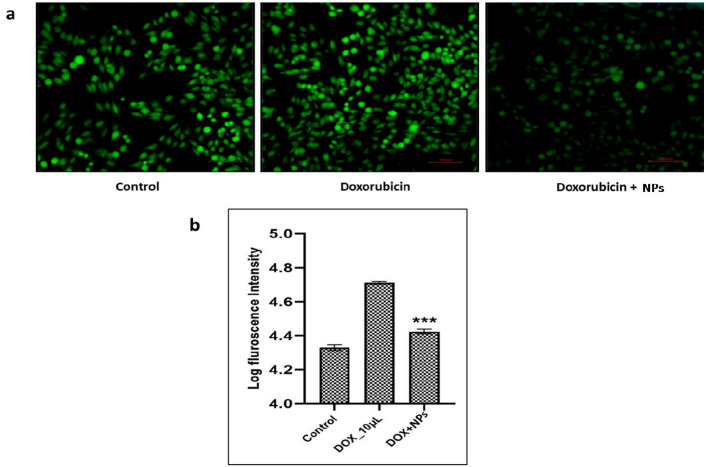
(**a**) Quenching effect in cells treated with nanoparticles compared to the control and DOX alone. (**b**) Graph showing the logarithmic fluorescence intensity of cells treated with DCFH-DA, *** *p* < 0.001.

T-47D breast cells were induced with exogenous ROS generator DOX and treated with nanoparticles to assess their antioxidant property. Oxidative stress was assessed using dichloro-dihydro-fluorescein diacetate (DCFH-DA). The fluorescence emission in DOX-treated samples was significantly high when compared with that of the control and treated samples (DOX + NPs). The decreased fluorescence emission in the treated sample indicates the quenching effect of the synthesized nanoparticles in the DCFH-DA assay (Figure 8a,b).

## 3. Discussion

In the last few years, chitosan application in medical research have significantly increased due to its high specificity, sensitivity, and reduced toxicity to mammalian cells [16,17]. Recently, chitosan application in the biomedical field for tissue engineering and drug delivery was also approved by the US FDA [18]. The interactive amino and hydroxyl groups of chitosan play a vital role in enhancing cell-to-cell adhesion, promoting low immunogenicity, and enacting bio-tolerability properties, which make chitosan an ideal candidate for biomedical applications [19,20]. However, studies indicate an enhanced synergistic effect of chitosan in biomedical usage when combined with metal nanoparticles [17].

In the present study, we synthesized silver nanoparticles using chitosan as a stabilizing and capping molecule and hydrazine as a reducing agent. During the synthesis process, silver added to the chitosan solution led to the formation of a silver core shell surrounded by the hydroxyl and amine groups of the chitosan, which was responsible for the capping and stability of the NPs [16]. The reducing agent hydrazine hydrate (N_2_H_4_·H_2_O) at lower concentrations (0.1–0.4%) aids in Ch-AgNPs formation by reducing silver nitrate to silver ions and eliminating nitrogen gas [21]. In the present study, a chitosan–acetic acid suspension was prepared prior to the initiation of the AgNPs synthesis process using hydrazine hydrate as a reducing agent; similarly, 0.2 g of chitosan was dissolved into 10 mL of 1% (*v*/*v*) acetic acid and used as a substrate for the preparation of silver nanoparticle–chitosan composite spheres [22]. Whilst in other studies chitosan pellets have been produced with optimal AgNPs using chitosan suspension in 0.2 M acetic acid [23], a similar methodology was also followed in our previous study, where hydrazine hydrate was used as a reducing agent for the preparation of chitosan copper nanoparticles [24]. In the present study, the zeta potential of synthesized nanoparticles was+19.2 mV, which is in corroboration with the available literature, in which the zeta potential value of silver nanoparticles was +36.0 mV, suggesting that the zeta potential value is highly dependent on the concentration of reducing or capping agent [22]. On the contrary, it has been stated that the zeta potential values of Ch-AgNPs range between − 0.0695 and − 0.225 mV, indicating the negative charge of zeta potential values [25]. The spectrophotometer peak recorded at 455 nm was known to be due to the surface plasmon resonance (SPR) of the NPs and its precursors in the dielectric environment of the solution [26]. Previous findings also support that the interaction of the amino and hydroxyl groups of chitosan polymer with silver ions occurs due to peak observed at this particular range [27]. Furthermore, the NPs synthesized in the current study had a positive surface charge which was directly related to the higher electrostatic repulsion between the NPs. The functional group of chitosan involved in the formation of Ag NPs was confirmed by FT-IR analysis, and the changes in the corresponding vibration band were related to the interaction between the Ag NPs and the chitosan amino and hydroxyl groups [28,29,30,31,32]. The stretching peak of an aliphatic primary amine (3344.41 cm^−1^) and a normal hydroxyl (−OH) group (3400−3200 cm^−1^) of chitosan coexisted due to their overlapping stretching frequencies, and this is well documented in the literature [33]. Further, bulk chitosan exhibited stretching peaks at 1384.11 cm^−1^ and 1647.83 cm^−1^ due to the presence of the -CONH_2_ group. The prepared Ch-AgNPs also showed peaks at 1384.26 cm^−1^ and 1640.93 cm^−1^, corresponding to the stretching of the functional group -CONH_2_ [28], suggesting the successful surface passivation of chitosan.

In addition, the XRD diffraction peak at 20.519° corresponds to chitosan, which confirms the successful passivation of Ag NPs with chitosan molecules; moreover, the observed 2θ values are in good agreement with reported values [25]. Further, FT-IR analysis showed a peak at 1565.66 cm^−1^ in the NPs compared to bulk chitosan, which may be due to amide functional involvement in the NP formation [34,35]. Thus, in our study, the sustainable fabrication of hybrid chitosan–silver nanoparticles was accomplished by using a simple and cost-effective reducing agent.

The application of antibiotics for the management of bacterial infections has been practiced for many years. However, in the present era, the use of conventional antibiotics has led to mediocre results due to the rapid development of resistant bacteria among nosocomial infections. In recent years, nanoparticles were found to be effective against several plant pathogens and are now garnering considerable attention for their use against clinical pathogens. In the present study, the synthesized Ch-AgNPs were screened for their antibacterial activity against two economically important clinical pathogens, namely *S. aureus* and *E. coli*, showing successful inhibition of both the pathogens. Similarly, our previous reports also support the antibacterial activity of silver nanoparticles against *S. aureus* and *E. coli* using the agar disk diffusion method [36]. Further, the role of chitosan in the release of metal nanoparticles at the infection site was highlighted in our previous study [24]. Likewise, another study also demonstrated that the silver nanoparticle-loaded collagen–chitosan dressing material could show 100% antibacterial activity at 3000 µg/cm^2^ [37].

Furthermore, synthesized Ch-AgNPs were used for an invitro cytotoxicity study, which are often used to characterize the biological response to nanoparticles. The results of such studies are helpful in identifying the toxic nature associated with nanoparticle exposure at higher concentration. HEK-293 is a robust and fast-growing cell linethat is highly reproducible and consistent. Moreover, HEK cells provide relevant information on human-specific responses to toxic substances, making them more suitable for assessing potential human health risks. Therefore, in the present study, cytotoxicity was carried out against normal HEK-293 cells at three different concentrations (1.5–6 μg mL^−1^) with appropriate controls. The toxicity study revealed that the IC_50_ value of the synthesized nanoparticles was 5.53 µg/mL, suggesting the biocompatible nature of the Ch-AgNPs when used at lower concentrations (<5.53 µg/mL). A study by Xue et al. also demonstrated that AgNPs are able to decrease the viability of HepG2 cells when exposed for 24 and 48 h at higher concentrations [38]. On the contrary, another study reported the variable toxicity of chitosan–silver nanocomposite particles, indicating that the toxicity of the nanoparticles is not only concentration-dependent but also highly dependent on the morphology of the nanoparticles [39]. Similarly, another comprehensive study on toxicity with various substrates using zebrafish embryos showed low-to-moderate toxicity, demonstrating that toxicity of is also dependent on the substrate composition used in the synthesized nanoparticles [40].

Several articles have reported the usage of chitosan-based nanoparticles in different forms, such as hydrogels, fibers, sponges, films, membranes and scaffolds, for wound healing assays [7,8,9,10,11,12,13]. However, information on chitosan-based silver nanoparticles in powdered form, which are quite effective in wound treatment, is very limited 15]. Therefore, the present study endeavored to evaluate the wound healing property of synthesized NPs on fibroblast cells (L292). The outcome of wound healing activity was promising, and we found a 97% recovery of wound with 0.55 µg/mL of NPs. The concentration used for wound healing activity was 10-times less than that of the IC_50_ value, which suggests the potential role of Ch-AgNPs in the management of wound infections. This enhanced activity might be due to the synergistic effect of chitosan and AgNPs, which accelerates the wound healing process by triggering inflammatory cells, macrophages, and fibroblast cells that help proliferate cells during the wound healing process [6,35,41]. Similarly, the available literature suggests that chitosan is one of the important biopolymers not only used in wound dressings for to enhance the wound healing process but also to inhibit bacterial infection in the wound area [42]. An adequate number of clinical trials (ongoing and completed) evaluating the safety and efficacy of chitosan for wound healing activity was also reported in another study [43]. Further, the wound healing activity of chitosan was found to be evident in both medical and veterinary domains [44].

In eukaryotic cells, ROS-induced apoptosis is generally escorted by the loss of mitochondrial membrane potential [45]. Oxidative stress can occur when ROS overcome the detoxifying ability of individual cells, which further leads to comorbidities such asinflammation, cancer, and neurodegenerative disease [45,46]. In our study, T-47D breast cells induced with exogenous ROS generator DOX increased florescence intensity 2.4-fold higher compared to a control. Meanwhile, T-47D breast cells treated with synthesized nanoparticles showed a reduced florescence intensity by 1.2-fold. The observed ROS scavenging property of synthesized Ch-AgNPs could be due to the hydroxyl (-OH) and amino (-NH_2_) groups in the biopolymer [47]. In addition, free amino groups present in the biopolymer (NH_2_) chitosan can react with OH to form stable macromolecule radicals, which are further degraded [48]. It was proven that the degraded products of biopolymer chitosan are non-toxic and cause no immunogenic reaction in the cells [49,50].

## 4. Conclusions

In the present investigations, chitosan solution was used as a stabilizing agent for the synthesis of silver nanoparticles, while hydrazine hydrate was used as reducing agent. Further characterization of the synthesized nanoparticles presented an absorption peak at ~455 nm with UV–Vis spectroscopy, and the morphology of the synthesized nanoparticles was found to be spherical, with a size of 15–30 nm, as confirmed by SEM and TEM analysis. The functional groups and crystalline nature of the synthesized nanoparticles were revealed by FTIR and XRD analysis. Furthermore, the chitosan–silver nanoparticles synthesized in the present study showed various biological activities, such as antibacterial activity against *S. aureus* and *E. coli*, wound healing activity, and antioxidant activity. Moreover, the MTT assay carried out in the present study also showed that the synthesized nanoparticles were non-toxic to host cells. Despite the usage of advanced antiseptics, wound infections remain a major concern in the health care sector due to the emergence of antibiotic-resistant microorganisms. Therefore, our study suggests that biocompatible polymer-based multifunctional silver nanoparticles could be more reliable for biomedical applications.

## 5. Materials and Methods

Silver nitrate (AgNO_3_, 99.98%), low-molecular-weight (LMW) chitosan, acetic acid (glacial, 99–100%), and hydrazine hydrate were procured from Sigma-Aldrich, St. Louis, MO, USA. Milli Q water was used throughout the experiment. Nutrient broth was purchased from Himedia Chemicals (Mumbai, India). *S. aureus* and *E. coli* cultures were grown and maintained at the Multi-Disciplinary Research Unit, Karnataka Medical College and Research Institute (KMCRI), Hubli, Karnataka, India.

### 5.1. Synthesis of Chitosan–Silver Nanoparticles (Ch-AgNPs)

The chitosan–silver nanoparticles were synthesized, with little modification of the previously reported procedures [22,24].

A chitosan suspension was prepared by dissolving 0.1 g of low-molecular-weight chitosan (75% deacetylated) into 10 mL of 1% (*v*/*v*) acetic acid with constant stirring for 18 h at room temperature. Further, chitosan–silver nanoparticles were synthesized using0.2% chitosan suspension and AgNO_3_ (1 mM) with constant stirring on a hot plate at 80 °C for 2 h; the reducing agent hydrazine hydrate (0.2%) was gradually added to this solution until the color changed to brown, indicating the conversion of AgNO_3_ to AgNps. The reduced nanoparticles (NPs) were centrifuged (8000× *g* for 10 min) with three washes. Further, they were dried in a hot air oven at 50 °C before they were subjected to physical and chemical characterization.

### 5.2. Characterization of the Nanoparticles

#### 5.2.1. Spectroscopic Studies

The synthesized NPs were initially observed using a UV-visible spectrophotometer (SP-UV 500DB; Uberlingen, Germany), with a 200–700 nm wavelength scan. Further, the NPs were subjected to Dynamic Light Scattering (DLS) to obtain the average particle size and polydispersity index using a high-performance particle size analyzer Z3000 (Nicomp, San Francisco, CA, USA). The surface charge was obtained by using zeta potential readings with a 7.4 pH graphite electrode at room temperature (SZ-100 particle size analyzer; Horiba; Koyoto, Japan). In addition, the bond vibrations of the functional groups involved in the NPs’ formation was recorded by Fourier transform infrared spectroscopy (FTIR; Nicolet 6700 Thermo Scientific, Waltham MA, USA) at 400–4000 cm^−1^. The cubic nature of the metal and chitosan, and silver passivation were recorded by X-ray diffraction studies using an AXS D8 X-ray diffraction system (Bruker; Billerica, MA, USA), with the Cu Kα line (1.5406 Å) employed to generate X-rays (40 kV/35 mA).

#### 5.2.2. Electron Microscopy Studies

The surface morphology of the NPs was studied by scanning electron microscopy(SEM) (Carl Zeiss EV018; Oberkohen, Germany). A thin smear of 10 μL (1:5; *v*/*v*) NCs suspension was placed on a microscopy stub to obtain a fine film, dried, and sputter-coated with a gold layer under high vacuum. Images were captured at a 7 mm working distance at different magnifications (10 K and 23 K). X-rays from the sample were captured by employing EDS with 5 kV energy. To obtain an accurate particle size, the NCs were subjected to transmission electron microscopy (TEM; Jeol Model JM 2100, Tokyo, Japan) and the images were recorded at different magnifications.

### 5.3. Antibacterial Activity of Ch-AgNPs

The antibacterial activity of the synthesized Ch-AgNPs was studied against Gram-positive *S. aureus* and Gram-negative *E. coli* by the broth dilution method [51]. *S. aureus* and *E. coli* were inoculated in 10 mL of nutrient broth (Hi-Media, Mumbai, India) and incubated at 37 °C for 16 h, and then they were sub-cultured in 10 mL of the same medium under the same conditions for 3 h to obtain the active growth phase. Different concentrations of NPs were serially diluted (100–0.2 µg mL^−1^) into each well; further, microbial inoculum (1:1000) was added and the final volume was made to 200 µL. Similarly, control antibiotics streptomycin and ampicillin were pipetted (100–0.2 µg mL^−1^) into the next two rows and serially diluted, and microbial inoculum was added to this. After 24 h of incubation at 37 °C, aqueous resazurin (30 µL) solution was added to each well and any change in color was recorded.

### 5.4. Cytotoxicity Assay of Ch-AgNPs by MTT

In the present study, normal HEK 293 (Human Embryonic Kidney) cells were used for the cyotoxicity assay, and they were procured from the National Chemical Laboratory (NCL), Pune. The cells were maintained in optimum condition at the Multi-Disciplinary Research Unit, KMCRI Hubli (minimum essential medium (MEM) supplemented with 10% fetal bovine serum at 37 °C, 5% CO_2_ with 100% relative humidity). The cells were centrifuged in a 15 mL tube at 1500 rpm for 5 min after trypsinization. The cell count of approximately 10,000 cells was adjusted using MEM, and 200 µL of the cell suspension was added into each 96-well plate, which was further incubated under the same conditions for 24 h. The media seeded with HEK-293 cells were treated with different concentrations (1.5, 3, and 6 µg/mL) of NPs and incubated under the same conditions for 24 h. Media without NPs and media with 6 µg/mL cisplatin were used as negative and positive controls, and the experiment was carried out three times. The averagesof the viable cells were plotted on agraph. After the incubation period, the rates of cell growth and inhibition was determined by 3-(4,5-dimethylthiazol-2-yl)-2,5-diphenyltetrazolium bromide (MTT). MTT reagent (0.5 mg/mL) was added to each well and incubated under the same conditions for 4 h. Inside the metabolically active cells, MTT was reduced to an insoluble, dark purple formazan. The medium was removed completely without disturbing the crystals formed, the formazan crystals were dissolved in 100 µL DMSO (dimethyl sulfoxide), and the plate was gently shaken to solubilize the formed formazan. The absorbance was measured using a microplate reader (Thermo Scientific, Multiskan Sky High Microplate Spectrophotometer, Waltham MA, USA) at 570 nm and 630 nm, and the percentage of cell inhibition was calculated using the following formula:

% cell inhibition = 100 − Absorbance (treatment)/Absorbance (Control) × 100

The concentration of NPs needed to inhibit cell growth by 50% (IC_50_) was then generated from the dose–response curve of the cell lines.

### 5.5. Wound Healing Property of Ch-AgNPs

A wound healing assay was carried out as per the protocol by [52], with minor changes. Normal fibroblast cells (L292 cells) were grown in DMEM with 10% FBS, and after confluence the cells were detached from the flask by trypsin, aspirated into a 15 mL falcon tube, and centrifuged at 1500 rpm for 5 min at RT. The known number of cells were suspended in 100 µL DMEM with supplement in a sterile 12-well plate and incubated for 24 h to reach ~100% confluence as a monolayer at 37 °C with 5% CO_2_. A scratch was made to the monolayer, and the wells were gently washed with DMEM to remove the detached cells; then, they were further washed with 1× phosphate-buffer saline (PBS). Media containing NPs were added (0.5 µg/mL) to each well and further incubated for 24 h. Ascorbic acid was used as a positive control. The wound gap distance was quantitatively evaluated using MagVision measurement tool at 4 × resolution. The cell migration rate was calculated using the following formula:

M_R_ = (W_i_ − W_t_)/T

Whereby M_R_ is the cell migration rate in micrometer scale (µM/h), W_i_ is the initial wound width (µM), W_t_ is the final wound width (µM), and T is the duration of migration (in hours).

### 5.6. Antioxidant Property of Ch-AgNPs

The antioxidantproperty of the Ch-AgNPs was evaluated by measuring the total reactive oxygen species (ROS) using a fluorescent dye 2′,7′-dichlorodihydrofluorescein diacetate (DCFH-DA) in adherent breast cancer cells by employing the Vanti et al. method, with somemodification [53].

This assay was carried out in 24-well sterile plates by using T-47D breast cells procured from the National Chemical Laboratory (NCL), Pune, India. In each well, cells were seeded (2 × 10^5^ cells) in Roswell Park Memorial Institute (RPMI) 1640 medium with supplements and incubated 48 h instandard conditions. Further, 48 h grown culture media were replaced with exogenous ROS generator doxorubicin (DOX) (10 μM) in RPMI medium and incubated for 4 h with the same conditions. Subsequent to the incubation period, media were replaced by 3 µg/mL NPs in 1×phosphate-buffer saline (PBS), control wells were incubated without NPs, and the experiment plate was incubated for 1h at standard conditions. After incubation, the medium from the each well was removed and the plate was washed twice with cold PBS (pH 7.4). To this, 500 μL of DCFH-DA working solution (10 μM) was added and the plate was incubated at 37 °C for 30 min in dark conditions. PBS from each well was removed, and RIPA lysis and extraction buffer (Thermo scientific) were added (300 µL) to each well; the plate was then incubated on ice for 5 min. Cell lysate was collected in tubes and cold centrifuged was conducted at 12,000 rpm for 10 min. The supernatant was carefully transferred (100 μL) to a black 96-well plate and the fluorescence was measured with 485 nm excitation and 530 nm emission wavelengths in a fluorometer (plate reader) (Tecan Infinite 200 PRO, Crailsheim, Germany). Fluorescence images were captured at 40× magnification using EVOS™ M7000 Thermo Fisher.

### 5.7. Statistical Analysis

All the experiments in the present study were carried out in triplicate, and the obtained results are presented as mean ± standard deviation. The analysis of data was carried out statistically using analysis of variance (ANOVA) with Prism version 8 software. Differences were deemed significant at the 0.001% level.

## Data Availability

Data are contained within the article.
